# Proposed highway in the Peruvian Amazon threatens vulnerable indigenous communities and natural protected areas

**DOI:** 10.1007/s13280-025-02175-z

**Published:** 2025-04-10

**Authors:** Brian M. Griffiths, David Dimitrie, Elizabeth Schierbeek, Edith Chinchilla Perez, Ellen Nirenblatt, Natalia Arcos Cano, Michael P. Gilmore

**Affiliations:** 1 The Earth Commons—Georgetown University’s Institute for Environment & Sustainability, 3700 O St. NW, Washington, DC 20007 USA; 2 OnePlanet, Inc., 6712 Wooden Spoke Rd, Burke, VA USA; 3 OnePlanet, Inc., Iquitos, Peru; 4 Detroit Zoological Society, Royal Oak, MI USA; 5 Department of Environmental Science & Policy, George Mason University, Fairfax, VA USA; 6 Nicholas School of the Environment, Duke University, Durham, NC USA; 7 School of Integrative Studies, George Mason University, Fairfax, VA USA

**Keywords:** Consultation, Convention 169, Development, Justice, Prior informed consent, Road

## Abstract

The conservation of biocultural diversity in the Amazon rainforest has become an international priority in the face of global change. Megadevelopment projects threaten conservation efforts and the rights of Indigenous communities to manage their ancestral lands. We examine the potential impacts of one proposed highway development project in the Peruvian Amazon, the Bellavista-El Estrecho Highway, on local and Indigenous communities and natural protected areas in the region. We found that zones of influence of the proposed highway eclipse 343 Indigenous communities of at least 33 000 Indigenous people of ten distinct cultures, 43 503 km^2^ of community land, and 26 210 km^2^ of natural protected areas including the entirety of the Maijuna–Kichwa Regional Conservation Area and the unique high terrace ecosystems it holds. Under international and national law in Peru, Indigenous communities who will be affected by megadevelopment projects like this highway must be engaged in prior consultation.

## Introduction

The Amazon is one of the most biologically and culturally diverse regions of the world, and the conservation of that diversity is an international priority. There are many threats to this biological and cultural diversity, including deforestation, oil drilling, overhunting, gold mining, and infrastructure projects such as roads and bridges (Gallice et al. 2019). Road development in the Amazon facilitates increased access to remote regions, allows settlers to migrate to these areas, and causes the rapid exploitation of natural resources in the well-known “fish bone” pattern (Arima et al. 2008). Road development in the Amazon has been directly linked to deforestation through fires, logging, and unsustainable hunting. In Peru, the devastating effects of road development are evident along the 2600-km interoceanic highway connecting Peru to Brazil (Oliveira et al. 2019).

A new megadevelopment project has arisen in a proposed highway from Iquitos, capital of the Loreto region of Peru, to San Antonio del Estrecho along the Putumayo River, which is the border with Colombia (Bellavista-El Estrecho Highway). This 188-km highway, planned by Provías Nacional of the Ministry of Transportation and Communications, is to be constructed in four phases: a bridge over the Nanay River, a road from the bridge to Mazán, on the Napo River, a ferry terminal to cross the Napo River, and a long stretch of highway (over 100 km) through the Maijuna–Kichwa regional conservation area (MKRCA) to Colombia. This project has been proposed to increase access to public services for rural populations (stated in Peruvian law Ley N^o^. 29 680; hereafter the Ley N^o^. notation describes Peruvian laws), and to encourage greater use of natural resources, facilitate increased tourism, reduce transportation costs, improve quality of life of local people, and increase employment.

We mapped the proposed route of the Bellavista-El Estrecho Highway along with three different zones of varying degrees of influence to quantify the potential impacts of the construction of the proposed highway in terms of number of Indigenous communities affected who would need to be consulted for the project, the area of natural protected areas, unique habitats, and more. One Indigenous group in particular, the Maijuna people, live in only four communities, all of which lie along the proposed highway route. We examined the livelihoods of the Maijuna to predict the impact the highway might have on their food security and traditional livelihoods.

## Methods

We mapped the proposed Bellavista-El Estrecho Highway route and its immediate 10-km zone of influence using QGIS (QGIS.org 2024); a conservative estimate of destruction, given that 83% of all deforestation in the Amazon has been seen to occur within 20 km of roads (Oliviera et al. 2007). We created 50-km and 150-km buffers surrounding the highway to examine the number and diversity of communities that would be affected by the project and therefore should be included in a prior consultation process under Peruvian law. These larger zones of influence were chosen because 95% of deforestation from a similar highway in Brazil was located within 50 km of the highway (Gallice et al. 2019), and 150-km zones should be considered for indirect impacts that fall under the ILO Convention 169 rules for prior informed consent (Ferrante et al. 2020). Alongside the zones of influence, we mapped Indigenous community lands that have been acknowledged by governments (IBC/CEPES-SICCAM 2024; IBC-SICNA 2024), local communities subject to the 2017 national census of Peru (National Institute of Statistics and Informatics (Peru) 2017), natural protected areas with approved legal status (UNEP-WCMC and IUCN 2024), and unique high terrace ecosystems which occur in the MKRCA (Gilmore et al. 2010). We estimate the number of Indigenous people impacted using stated population sizes of Indigenous communities, acknowledging this is likely a severe underestimate given Indigenous people also live in communities which are unrecognized, and not every household participates in the census.

## Results

**Fig. 1:**
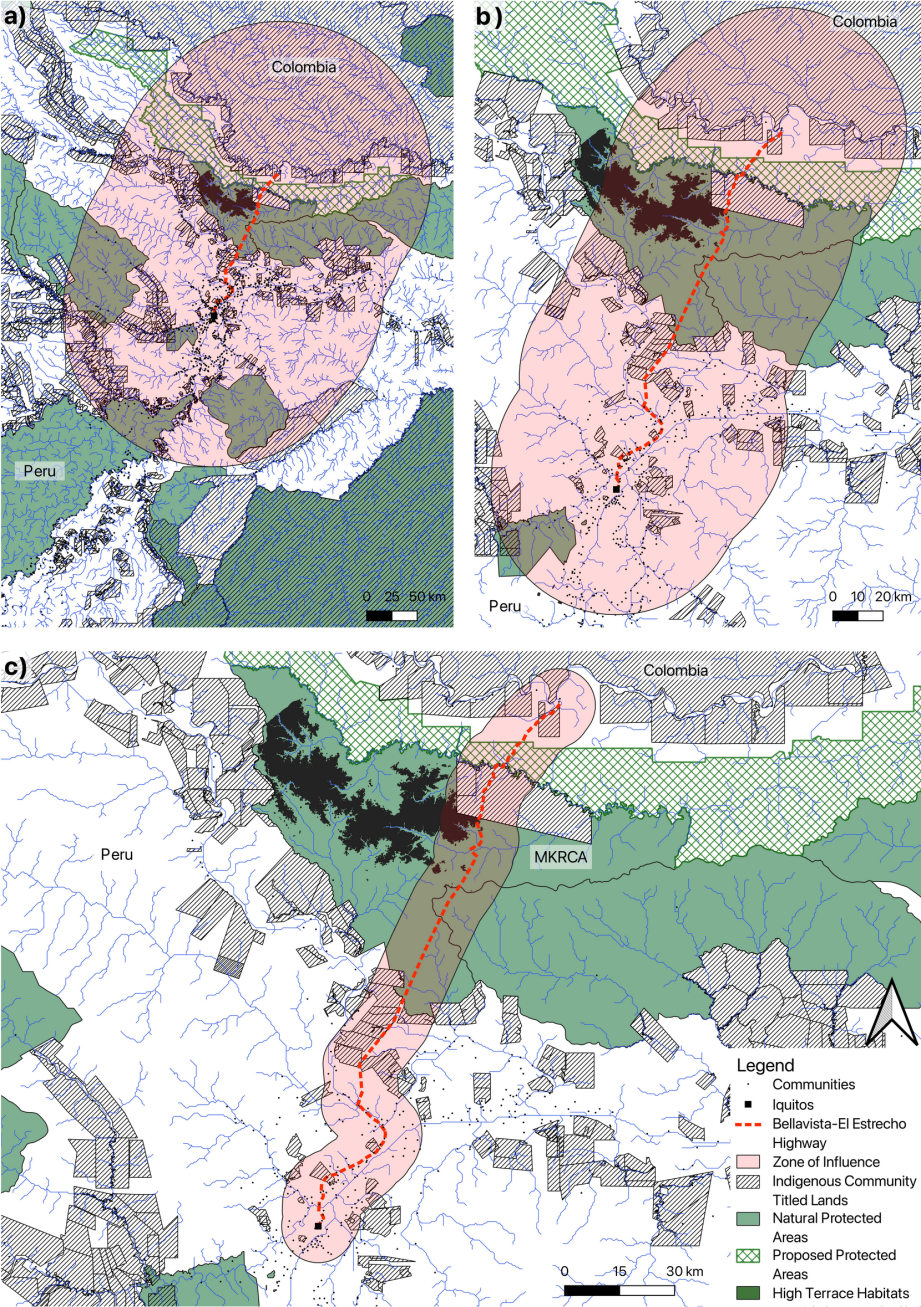
Proposed route of the Bellavista-El Estrecho Highway and the Indigenous communities and titled lands that lie in the **a** 150 km, **b** 50 km, and **c** 10-km zones of influence of the proposed road. Also shown are high terrace ecosystems discovered in the Maijuna–Kichwa Regional Conservation Area (MKRCA), a unique habitat type that is particularly vulnerable. Map lines delineate study areas and do not necessarily depict accepted national boundaries

The 150-km zone of influence from the proposed highway encompasses the titled lands of 343 Indigenous communities made up of ten distinct ethnic groups: the Bora, Ocaina, Iquito, Kichwa, Huitoto, Yagua, Maijuna, Achuar, Mayoruna, and Cocama-Cocamilla, each of which has their own language and traditional culture. Together, these communities are estimated to have a population of 13 171 Indigenous people who hold title to 43 504 km^2^ of land in the zone of influence (Table 1). About 181 063 non-Indigenous people in 336 communities also live in this zone. The 150-km zone of influence includes the entirety of the MKRCA and its unique high terrace forest ecosystem (642 km^2^) (Fig. 1a). Notably, the zone of influence also encompasses major portions of the Predio Putumayo Indigenous Reserve of Colombia, which contains the largest contiguous area of rainforest remaining in Colombia, and two proposed protected areas which are under consideration by the Peruvian government.

**Table 1:** Estimated impacted areas and populations from road construction based on three zones of influence at different distances from the proposed highway route

Impact	Zone of influence distance (km)		
	10	50	150
Indigenous population	2 787	7 032	33 736
# Indigenous communities	25	75	343
Non-Indigenous population	122 425	157 244	181 063
# Non-Indigenous communities	85	290	336
Community land area (km^2^)	796	6605	43 504
Natural protected areas (km^2^)	1097	5612	26 210
High terrace ecosystem (km^2^)	59	494	642
% of MKRCA land	27.2	90.3	100

The 50-km zone of influence from the proposed highway overlaps with 75 Indigenous communities made up of six distinct ethnic groups: the Yagua, Huitoto, Kichwa, Bora, Iquito, and Maijuna. Together, these communities have an estimated population of 7 032 Indigenous people (Table 1). We estimate 157 244 non-Indigenous people live in this area. The 50-km zone of influence, which may describe up to 95% of deforestation associated with the highway (Gallice et al. 2019), encompasses 5612 km^2^ of natural protected areas and 6605 km^2^ of community land (Table 1). The 50-km zone of influence covers 90.26% of the total land area of the MKRCA, and 76.91% of the total area of unique MKRCA high terrace forest habitat (Fig. 1b).

The proposed highway and 10-km zone of influence pass directly through titled lands of four Maijuna communities, three Yagua communities, 13 Kichwa communities, two Bora communities, and three Huitoto communities. The proposed road passes within 20 km of all three additional Maijuna communities (Fig. 1c). The area within the 10-km zone of influence, 914 km^2^ of MKRCA land, including 58.62 km^2^ of MKRCA high terrace forest habitat, is expected to be almost completely deforested by the direct construction of the highway and an immediate influx of settlers. The zone of influence includes 796 km^2^ of community land, with an estimated Indigenous population of 2 787 people (Table 1). We estimate 122 425 non-Indigenous people live in the 10-km zone of influence (Table 1).

### Loss of biodiversity and globally significant carbon stores

The biodiversity of this region, including genes, species, and habitats, is a full sample of the megadiversity of the Amazon rainforest. The region along the path of the highway is a mosaic of upland and flooded forests. Upland forests in the MKRCA hold high terrace ecosystems which lie directly in the path of the highway. High terraces––a unique, previously unknown habitat––shelter new, rare, and specialized flora and fauna species (Gilmore et al. 2010). These forests are also habitats for endangered and vulnerable species (IUCN 2024), including the tapir (*Tapirus terrestris*), jaguar (*Panthera onca*) and other felids, white-lipped peccary (*Tayassu pecari*), large-bodied primates like the red howler monkey (*Alouatta seniculus*) and woolly monkey (*Lagothrix lagotricha*), and more (Griffiths et al. 2022). The loss of these habitats will result in the local extirpation of many of these species, and may even facilitate the invasion of alien species of plants and animals.

Flooded forests cover vast swathes of the landscape in palm swamps, which hold relatively high carbon sequestration capacity. Deforestation and degradation of these peat lands will result in increased methane emissions and the loss of carbon storage capacity of these forests, turning a carbon sink into a carbon source. Conservation of these carbon stocks is of international concern to mitigate the effects of global climate change, and offer an opportunity for Peru to meet its agreed climate goals of limiting emissions by 30% by 2030, and conserving forested ecosystems (Gobierno del Perú 2020).

### Regional impacts on livelihoods and food security

Local communities depend on regional protected areas for food security, particularly fish and game meat for dietary protein. Rural communities in the Peruvian Amazon harvest hundreds of thousands of kilograms of game meat annually (Mayor et al. 2021). The trade of game meat within communities and with the regional market is extensive, with hunters supporting neighbors’ food security through social gifting systems (Griffiths and Gilmore 2022) and urban consumers through vast supply chains (Mayor et al. 2021). Hunting remains the largest single source of income for some communities. Many commonly hunted large mammals cannot sustain a healthy population under hunting or logging pressure without a large source area nearby (Rija et al. 2020).

The sale of non-timber forest products is also a critical source of income, particularly fruit from the *aguaje* tree (*Mauritia flexuosa*), which has an enormous regional supply chain. The *aguaje*, a dioicous palm forming carbon-rich peat swamps, shifts to all-male tree stands that eventually result in a collapse of the system when female trees are cut down (Gilmore et al. 2013). Many rural communities also depend upon rich plant genetic diversity for agriculture and traditional medicines, which constitute substantial value (United Nations 1992). As observed throughout the Amazon, settlers along newly constructed roads unsustainably harvest game, fish, and other non-timber forest products from surrounding forests and protected areas, negatively affecting biodiversity, local livelihoods, and food security (Gallice et al. 2019).

### Loss of local and indigenous culture

The Amazon rainforest makes up vast portions of the ancestral lands of Indigenous communities in the Loreto region, and holds great spiritual importance for those communities. For example, many cultures have habitat classification systems rooted in traditional ecological knowledge that are often much more nuanced than Western scientists’ classifications (Gilmore et al. 2010). Many cultures, including the Maijuna, have traditional narratives on a multitude of species, from primates to tapirs, and even *aguaje*. The loss of these culturally salient organisms and habitats, and the invasion of colonists will result in the rapid erosion of traditional cultural practices and traditional ecological knowledge, much of which promotes the sustainable use of natural resources. The cultural ecosystem services provided by these organisms and habitats are of unquantifiable value to the cultural diversity of the Amazon Rainforest.

## Conclusion

**Fig. 2:**
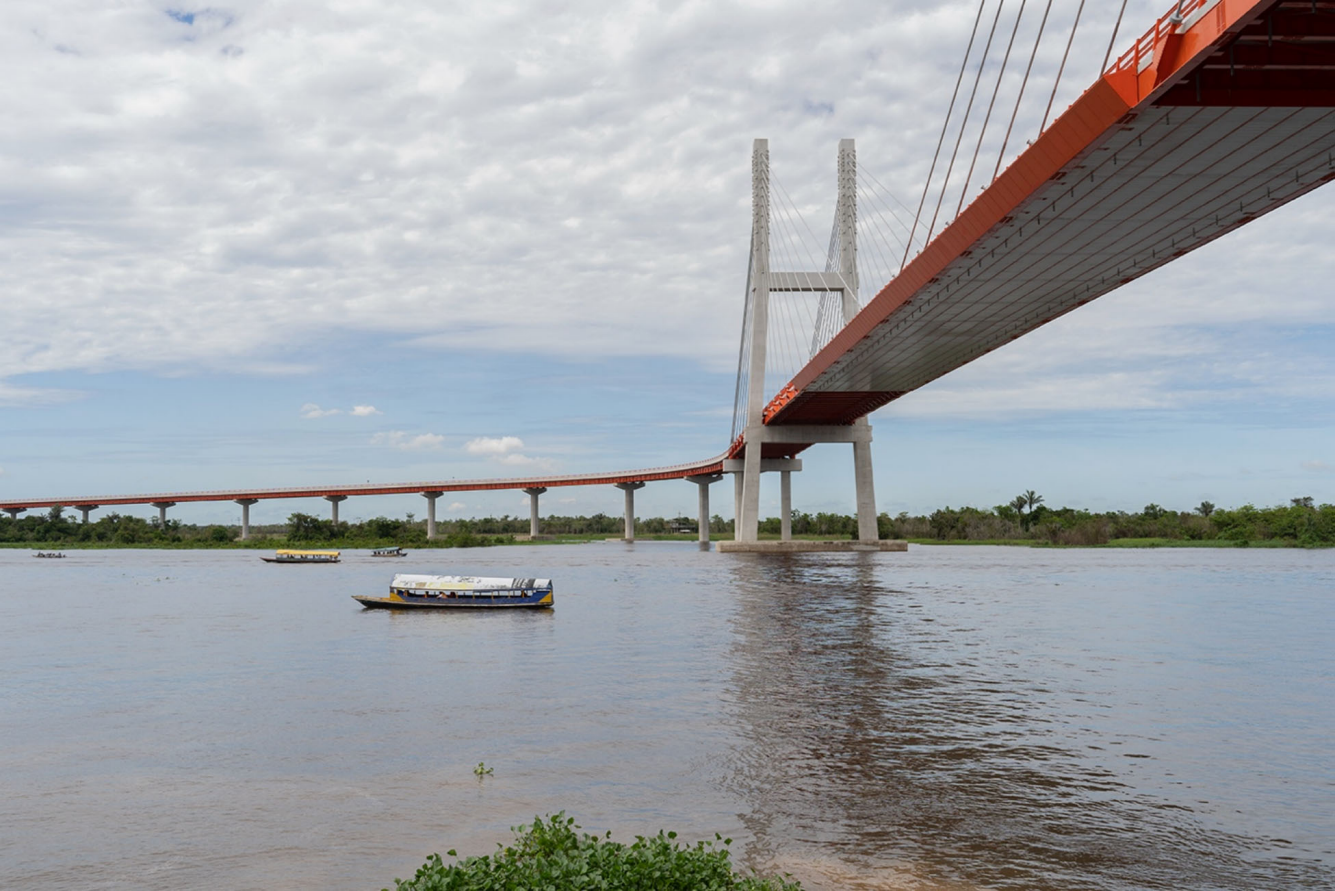
Completed bridge over the Nanay River as Phase 1 of the proposed Bellavista-El Estrecho Highway. Photograph by Enrique Redondo Navarro

Convention 169 of the International Labour Organization (ILO), the Indigenous and Tribal Peoples Convention, signed by Peru in 1994 and adopted into Peruvian law in 2011 (N^o^. 27 446, 29 325, and 29 785), requires the consultation of Indigenous communities that would be directly or indirectly affected by development projects (International Labour Organization (ILO) 1989). We demonstrate that Indigenous communities within 50 km of the road will be directly impacted by the highway, and those within 150 km indirectly impacted. The impacts of road construction on biodiversity, local livelihoods, and traditional ecological knowledge have been well documented. The Bellavista-El Estrecho Highway is likely to impact 343 Indigenous communities, and tens of thousands of square kilometers of community land and natural protected areas which are critical for local livelihoods and biodiversity. Phase 1 of the Bellavista-El Estrecho Highway has already been completed, and the longest bridge ever constructed in Peru now spans the Nanay River from the city of Iquitos (Fig. 2); however, no prior consultation has been completed by the Peruvian government to date (Gamboa and Quispe Dávila 2023; ODGS and MTC 2024).

Local people in this region are already accustomed to the use of waterways for travel between communities and the city of Iquitos. We recommend that the Peruvian government invest in existing fluvial transport infrastructure to make public transit by boat accessible for all local people, bringing greater connectivity without sacrificing the socioecological integrity of the region. Because of the topography of the northeastern region of Loreto, access to Colombia via river is difficult and lengthy. We suggest that if a shorter route to Colombia is sought, to promote international trade and movement, the Peruvian government could explore an alternate route for the highway which has fewer impacts to people and natural areas. For example, the Maijuna have suggested restarting a previous road project that lies northwest of the MKRCA, which is much shorter, does not threaten nearly as many people or protected areas, and had already begun decades before (FECONAMAI and FECONAMNCUA 2020).

## Data Availability

This manuscript uses only publicly available data that has been appropriately cited.
