# Correction: Proposed highway in the Peruvian Amazon threatens vulnerable indigenous communities and natural protected areas

**DOI:** 10.1007/s13280-025-02311-9

**Published:** 2025-11-26

**Authors:** Brian M. Griffiths, David Dimitrie, Elizabeth Schierbeek, Edith Chinchilla Perez, Ellen Nirenblatt, Natalia Arcos Cano, Michael P. Gilmore

**Affiliations:** 1 The Earth Commons—Georgetown University’s Institute for Environment and Sustainability, 3700 O St. NW, Washington, DC 20007 USA; 2 OnePlanet, Inc., 6712 Wooden Spoke Rd, Burke, VA USA; 3 OnePlanet, Inc., Iquitos, Peru; 4 Detroit Zoological Society, Royal Oak, MI USA; 5 Department of Environmental Science & Policy, George Mason University, Fairfax, VA USA; 6 Nicholas School of the Environment, Duke University, Durham, NC USA; 7 School of Integrative Studies, George Mason University, Fairfax, VA USA

**Correction: Ambio 2025, 54:1103–1108** 10.1007/s13280-025-02175-z

In the original published article, the shapefile for community locations (*centros poblados*) was incomplete, and did not match the associated shapefile for Indigenous titled lands, resulting in an underestimation of the number of Indigenous communities and Indigenous people who may be impacted by the proposed highway.

The incorrect and correct summaries of the data in Table 1 are provided in this correction.

The original article has been corrected.

Incorrect version of Table 1:

**Table:** 

Impact	Buffer Distance (km)		
	10	50	150
Indigenous Population	1023	2816	13 171
# Indigenous Communities	6	22	99
Non-Indigenous Population	124 189	161 460	201 628
# Non-Indigenous Communities	104	343	580
Community Land Area (km^2^)	796	6605	43 504
Natural Protected Areas (km^2^)	1097	5612	26 210
High Terrace Ecosystem (km^2^)	59	494	642
% of MKRCA Land	27.2	90.26	100

Correct version of Table 1:

**Table:** 

Impact	Zone of Influence Distance (km)		
	10	50	150
Indigenous Population	2787	7032	33 736
# Indigenous Communities	25	75	343
Non-Indigenous Population	122 425	157 244	181 063
# Non-Indigenous Communities	85	290	336
Community Land Area (km^2^)	796	6605	43 504
Natural Protected Areas (km^2^)	1097	5612	26 210
High Terrace Ecosystem (km^2^)	59	494	642
% of MKRCA Land	27.2	90.26	100

